# Health and Wealth

**Published:** 1856-05

**Authors:** 


### HEALTH AND WEALTH.

Most persons have a kind of spite or grudge against rich people, the foundation of which we presume is in Envy, one of the very meanest feelings of our nature. "He has more than his share, more than he can use, and I have less, my family are starving," said a poor fellow one day when he was asked if he had anything to say in mitigation of his sentence for a paltry theft from his wealthy neighbor's premises, "and people ought to be made to divide." "But suppose there was an equal division," replied the judge, who for a moment felt willing to humor the prisoner's absurdity, "your idle habits and the industry of your neighbor would soon make as wide a difference between your respective conditions as there exists at present."

"Very true, your honor," said lazy, "then we would divide again."

Just as ridiculously absurd and one-sided are many of the sentiments entertained by the poor towards their more thrifty fellow citizens. But the rich can afford the indulgence of these and kindred feelings against them.

Many of us have a sufficient want of magnanimity to cherish an inexpressed chuckle of gratification, at the intelligence that some notably rich family has met with some sudden calamity, personal, domestic or pecuniary; and sometimes, the less cautious out-slip such an expression as "served them right." "Ah! his wealth could'nt save him." "He ought to have trouble." "Nothing more than he deserves." How infinitessimally small is the poor human heart sometimes, in some of its phases. Of a multitude of wrong impressions about the rich, we single out one, more particularly appropriate to the pages of a Journal of Health. It is this, that one of the penalties of wealth is disease; this is not so, the rich are not more sickly as a class, than the poor; they are not as much exposed to the causes of disease as the poor are, their lives are more equable, less subject to great exposures whether to the extremes of labor, or of active effort or of heat and cold, and privation and hunger. Statistics in European countries plainly show, as exhibited in a previous number of our Journal, that the average age of the well to do in the world is greater, by quite a number of years, than that of the struggling poor. If the price of health were poverty, then it is a bootless endeavor to strive for the means of securing comfortable dwellings, and abundant fuel and clothing, and provision for the cheerless winter time. Especially is it true in large towns and in cities, that it is the children of the poor, who from want and neglect fill our grave-yards: often does the weekly mortuary report show the appalling fact that more than half of all who die, are young children, and a more minute examination of the list shows to the physician that the very large proportion of such deaths, are those which have their origin in exposure, and want and cold. How few of our comparatively very rich men die short of the sixties. In New Orleans, where exposures at certain seasons are so fatal, the very rich live to an old age, as witness McDonough, and Touro, and Fisk and Wilder, and a long list of others. Their wealth made exposures less necessary, and enabled them to take the world easy, a prophylactic which counteracts many a drunken bout, many a midnight carousal, many a gourmandic dinner; as witness too the Lords, and Bishops, and Chancellors, and Dukes of England, who so often measure to the eighties, and at last, like the "*Iron Duke*" die with their harness on, in the full performance of their civil duties, to which results we believe they are mainly indebted to their wealth, which affords to them the comforts of life without embarrassment, while it gives them time for all things, relieving them from that weary, wearing, wasting away, which is the inevitable result of our Yankee hurry, time and means to roll in the carriage, to drive in the phæton or gallop on the horse over hills and dales, and far away. Our word for it, half of all the glorifications of poverty and its advantages, which so often help to turn a sentence or to fill out a line, are mere balderdash, the coinings of fledgelings of the quill, or of brandied brains. We never could see any advantages in poverty, which intelligent wealth could not compass. Poverty, *per se*, is disreputable to any man, just as wealth of itself, is creditable to its possessor, being, as it is, *prima facie* evidence of long years of industrious economies and courageous self denials. That worthy people may be poor, and that unworthy people may be rich, we do not contravene; we are speaking of the rules, not the exceptions. In our opinion, those who reprobate the rich so glibly, are a set of poor lazy good for nothings, whose idolatry is their ease, *whose god is their belly, and who glory in their shame.* Who pretends that the poverty of a nation is not its crime, and reasoning from the greater to the less, from the masses to the individuals, is not particularly unsafe in this connection. It is the care of to-morrow, the gnawing, corroding anxieties for the future, which eat away the health and life of multitudes. The rich man and the slave are wholly free from this everlasting worm, while in its stead, there is an abiding composure and quietude, worth more than all medicine, and to this we attribute in great part, the truth of one of the revelations of the last census, that thirty-three and a third per cent. more of blacks were reported to have died of old age than of whites; although there are seven whites where there is one negro.

There is another interesting similarity in the life of the rich man and the slave. While the fear of want troubles neither of them, their previous lives, although from very different causes, bear a striking resemblance, their lives have been lives of active industry, lives of temperance and self-denial, compulsory as to the slave, but from choice, habit, principle, on the part of the white Dives. We are speaking specially, be it remembered, of those who have made their own fortunes.

From the laboratory of the Doctor then, we issue this formula, divested of all its hieroglyphical technicalities, and issue it too, with singular confidence for universal good, to wit:

If you desire to live long in ease and comfort, free from grunts and groans, and aches and pains; if you would have a countenance of genial sunshine, instead of vinegar, if you would be overflowing with risibilities, instead of being racked with Rheumatics; get rich, by spending your youth in temperate industries and prudent economies, having in view the wise and kindly expenditure of your wealth, in a healthful old age.

